# Uncovering Diagnostic Value of Mitogenome for Identification of Cryptic Species *Fusarium graminearum Sensu Stricto*

**DOI:** 10.3389/fmicb.2021.714651

**Published:** 2021-08-31

**Authors:** Joanna Wyrębek, Tomasz Molcan, Kamil Myszczyński, Anne D. van Diepeningen, Alexander A. Stakheev, Maciej Żelechowski, Katarzyna Bilska, Tomasz Kulik

**Affiliations:** ^1^Department of Botany and Nature Protection, University of Warmia and Mazury in Olsztyn, Olsztyn, Poland; ^2^Department of Bioinformatics, Institute of Biochemistry and Biophysics, Polish Academy of Sciences, Warsaw, Poland; ^3^Molecular Biology Laboratory, Institute of Animal Reproduction and Food Research, Polish Academy of Sciences, Olsztyn, Poland; ^4^Biointeractions & Plant Health, Wageningen Plant Research, Wageningen, Netherlands; ^5^Shemyakin and Ovchinnikov Institute of Bioorganic Chemistry, Russian Academy of Sciences, Moscow, Russia

**Keywords:** *Fusarium graminearum sensu stricto*, *Fusarium graminearum* species complex, homing endonucleases, introns, mobile genetic elements, identification

## Abstract

Fungal complexes are often composed of morphologically nearly indistinguishable species with high genetic similarity. However, despite their close relationship, they can exhibit distinct phenotypic differences in pathogenicity and production of mycotoxins. Many plant pathogenic and toxigenic fungi have been shown to consist of such cryptic species. Identification of cryptic species in economically important pathogens has added value in epidemiologic studies and provides opportunities for better control. Analysis of mitochondrial genomes or *mitogenomics* opens up dimensions for improved diagnostics of fungi, especially when efficient recovery of DNA is problematic. In comparison to nuclear DNA, mitochondrial DNA (mtDNA) can be amplified with improved efficacy due to its multi-copy nature. However, to date, only a few studies have demonstrated the usefulness of mtDNA for identification of cryptic species within fungal complexes. In this study, we explored the value of mtDNA for identification of one of the most important cereal pathogens *Fusarium graminearum sensu stricto* (*F.g.*). We found that homing endonucleases (HEGs), which are widely distributed in mitogenomes of fungi, display small indel polymorphism, proven to be potentially species specific. The resulting small differences in their lengths may facilitate further differentiation of *F.g.* from the other cryptic species belonging to *F. graminearum* species complex. We also explored the value of SNP analysis of the mitogenome for typing *F.g.* The success in identifying *F.g.* strains was estimated at 96%, making this tool an attractive complement to other techniques for identification of *F.g.*

## Introduction

*Fusarium graminearum sensu stricto* (*F.g.*) ranks number 4 in the top 10 most important and best-studied species that cause diseases on agriculturally important plants (Dean et al., 2012). The fungus is often involved in two diseases of cereals: fusarium head blight (FHB) of wheat and barley and fusarium ear rot (FER) of maize, which lead to major losses for grain production worldwide. Besides yield reduction, *F.g.* is able to produce mycotoxins, among which trichothecenes pose a serious hazard to human and animals (Dong et al., 2020; Yao et al., 2020). *F.g.* belongs to the monophyletic fungal complex referred to as *F. graminearum* species complex (FGSC). This complex includes 16 genetically characterized cryptic species (Sarver et al., 2011), several of which are involved in cereal diseases in certain agricultural areas. The species are difficult to identify by morphological characters and often share a high DNA sequence similarity (Walkowiak et al., 2016). However, despite their close relationship, FGSC species and even strains within species can exhibit distinct phenotypic differences in pathogenicity and mycotoxin production, while some strains lack pathogenicity on certain hosts (Jianbo, 2014; van der Lee et al., 2015; Walkowiak et al., 2016).

The increasing number of genomic sequences that are deposited in public databases has enabled research in genomic features that contribute to phenotypic variation and niche specialization within FGSC. Genome comparisons revealed significant divergence between them that can be mostly linked to single-nucleotide polymorphisms (SNPs), insertions/deletions (indels), and gene content variation, which is species specific or fixed in certain populations. Comparative genomics provides insights into the evolutionary processes contributing to pathogen divergence at both the macroevolutionary and microevolutionary scales (Walkowiak et al., 2016; Kelly and Ward, 2018).

Besides nuclear genome analyses, analysis of fusarium mitogenomes may provide useful data to study phylogenetic relationships and evolution. Despite high conservation, mitogenomes of fungi within complexes often exhibit a significant degree of polymorphism (Brankovics et al., 2017, 2020). This variation largely results from the irregular distribution of mobile genetic elements (MGEs) such as introns and associated homing endonucleases (HEGs; Basse, 2010; Joardar et al., 2012; Pogoda et al., 2019). MGEs comprise a substantial fraction of fungal mitogenomes and display a different evolutionary history from other mitochondrial loci (Guha et al., 2018; Kolesnikova et al., 2019). Their mosaic distribution throughout evolution can be explained with the “aenaon” model, which combines “intron-early” evolution enriched by “intron-late” events through recombination involving vertical and horizontal gene transfer (Megarioti and Kouvelis, 2020). Notably, variation in MGE content appears to be dependent on taxonomic sampling. This variation can be highly conserved, indicating a low mobility of ancestrally acquired MGEs (Kulik et al., 2020b). In contrast, more recent enrichment events result in increased mosaicism of MGE patterns that can be observed at the population level (Wolters et al., 2015).

Fungi of the genus *Fusarium* display a high variation in MGE content, from MGE-poor to MGE-rich mitogenomes (Losada et al., 2014; Brankovics et al., 2017). Our previous study performed on a large collection of *F.g.* strains has shown that the mitogenome of this fungus is MGE rich and mainly includes introns from the group I intron family, which harbor either LAGLIDADG or GIY-YIG HEGs. Comparison of mitogenomes of geographically diverse *F.g.* strains did not reveal any population-specific profiles, thus supporting the hypothesis on ancestral acquisition of HEGs. Assuming their early acquisition and low mobility, recently diverging cryptic species may thus share a similar content of MGEs (Kulik et al., 2020b). This hypothesis will be tested using mitogenomes obtained in this study.

Variation in mitogenomes of closely related fungi may also be detected by phylogenetic conflict between single-gene trees. This phenomenon was discovered among and within members of closely related fungal complexes: the *Fusarium fujikuroi* species complex (FFSC) and *Fusarium oxysporum* species complex (FOSC; Brankovics et al., 2020). Conflicting tree topologies indicate incomplete lineage sorting from ancestral polymorphism or more recent interspecies gene flow since both can result in similar phylogenetic patterns (Fourie et al., 2013, 2018). The phylogenetic discordance complicates interpretation of phylogenetic reconstructions and largely limits clustering-based identification approaches (Wu et al., 2018).

Besides its evolutionary value, mitochondrial DNA (mtDNA) is promising in the diagnostics of fungi, especially with regard to its multi-copy nature, which facilitates its high recovery and amplification success (Santamaria et al., 2009). Both MGE content variation and SNP in mitogenomes could be used for identification purposes of fusaria (Wu et al., 2019; Kulik et al., 2020b). However, discriminating cryptic species based on mitochondrial sequences is challenging due to the high degree of conservation, and detecting species-specific sites requires screening of a large set of genomic data to differentiate between intraspecific and interspecific variation (Kulik et al., 2015, 2020a).

The analysis of SNPs provides an excellent tool for identifying and typing species (Uelze et al., 2020a). In general, SNPs display relatively low mutation rates and are evolutionarily stable (Leekitcharoenphon et al., 2012). Usually, SNPs are identified by mapping sequence data from individual strains against a closely related reference genome. This type of analysis relies on the generation of a certain number of SNPs. It includes all studied genomes, including the reference genome (Uelze et al., 2020b).

The aims of this study are (1) to explore whether MGE polymorphisms can be used for identification of *F.g.*, (2) discover the value of SNP analysis for typing *F.g.*, and (3) confirm the utility of mt-based phylogenetic approach for the determination of *F.g.* identity.

In total, 122 strains were analyzed: 88 strains of *F.g.* and 34 strains representing other members of the FGSC. We discovered that HEGs present in mitogenomes of the FGSC display small indel polymorphism, which facilitates recognition of *F.g.* We also showed that whole-mitogenome SNP analysis enables typing of *F.g.* with 96% confidence, which can make this tool a valuable complement to other diagnostic tools for *F.g.* A phylogenetic analyses based on core mitochondrial genes showed different topologies in the reconstructed phylogenetic trees and did not cluster strains of *F.g.* into single species-specific clades, which neglects the value of this approach for determination of species identity.

## Materials and Methods

### Fungal Strains

In total, 122 strains were analyzed in this study, with 88 *F.g.* strains and 34 strains representing all known cryptic species from the FGSC complex: *Fusarium acaciae-mearnsii* (three strains), *Fusarium aethiopicum* (two strains), *Fusarium asiaticum* (three strains), *Fusarium austroamericanum* (two strains), *Fusarium boothii* (three strains), *Fusarium brasilicum* (two strains), *Fusarium cortadariae* (three strains), *Fusarium gerlachii* (two strains), *Fusarium louisianense* (two strains), *Fusarium meridionale* (one strain), *F. meridionale* × *F. asiaticum* hybrid strain (one strain), *Fusarium mesoamericanum* (two strains), *Fusarium nepalense* (two strains), *Fusarium ussurianum* (two strains), *Fusarium vorosii* (three strains), and strain CBS 123663, which lacks a Latin binomial. All strains used in this study are included in Supplementary File S1.

### DNA Extraction and Sequencing

DNA from fungal strains was extracted from 0.1g of mycelium harvested from PDA medium with the use of the Quick-DNA Plant/Seed Miniprep Kit (Zymo Research, Irvine, CA, United States) and Genomic Mini AX Food (Gdynia, Poland) according to the manufacturer’s protocols. Genome libraries were constructed using either a TruSeq DNA PCR-free library preparation kit (Illumina, San Diego, CA, United States) or a KAPA HyperPlus Kit (Roche Sequencing Solutions, Pleasanton, CA, United States). Whole-genome sequencing was performed by Macrogen (Seoul, South Korea). An Illumina HiSeq X 10 platform was used to sequence the genomes using a paired-end read length of 2×150bp with an insert size of 350bp.

### Assembly and Annotation of Mitogenomes

To perform *de novo* assembly of fungal mitogenomes from whole-genome data, NOVOPlasty 2.7.2 (Dierckxsens et al., 2017) was used. HEGs were identified using NCBI’s ORF Finder, Blast+ (v. 2.9.0; Johnson et al., 2008), and Geneious Prime software (Biomatters Ltd., New Zealand). Annotations were performed using MFannot, InterPro (Mitchell et al., 2015), CD-Search (Marchler-Bauer and Bryant, 2004), and Geneious Prime software (Biomatters Ltd., New Zealand). Annotation of tRNA genes was improved using tRNAscan-SE (Pavesi et al., 1994). Annotations were manually verified and corrected. Complete mitogenomes were deposited in the NCBI database under the GenBank accession numbers given in Supplementary File S1.

### Exploration of MGE Diversity

A total of 122 mitogenomes were compared through multiple sequence alignments. The analysis was performed with progressive Mauve (Darling et al., 2010) implemented in Geneious Prime software to examine the distribution of MGEs in the mitogenomes.

### SNP Analysis

SNP analysis was performed on the interactive web-based platform Edge bioinformatics.^1^ SNPs were detected in multiple comparisons of assembled mitogenomes of the strains to the reference whole genome of PH-1 of *F.g.* (accession number: MH412632). Eight mitogenomes from closely related morphospecies (*Fusarium culmorum*, *Fusarium cerealis*, and *Fusarium pseudograminearum*) were also included in the analysis.

### Phylogenic Analysis

Phylogenetic analyses were performed on single-gene alignments of coding sequences of 15 core mitochondrial genes (*cox 1–3*, *cob*, *atp6*, *atp8*, *atp9*, *nad1-6*, *nad4L*, and ribosomal *rnl* gene) from 130 strains: 122 mitogenomes of FGSC and eight mitogenomes from closely related morphospecies (*F. culmorum*, *F. cerealis*, and *F. pseudograminearum*). Coding sequence data (CDS) from 15 core mitochondrial genes was first aligned using MAFFT software (v.7.453; Katoh and Toh, 2010) with default settings. Single-gene phylogenetic analyses were performed for *atp9*, *cox1*, and *cox2*. The remaining single-gene alignments were excluded for further phylogenetic analyses due to low sequence polymorphism. The best partition schemes and corresponding substitution models were estimated using PartitionFinder2 (Lanfear et al., 2016). Afterwards, based on the alignment and obtained models, maximum likelihood analysis was conducted using IQ-TREE 2.0.3 with 1,000 ultrafast bootstrap (Minh et al., 2020). *F. pseudograminearum* was used as an outgroup.

To reveal variation in mitochondrial CDS, core mitochondrial genes were extracted and aligned separately using MAFFT software (v.7.453; Katoh and Standley, 2013). Gene polymorphism analyses were conducted for each CDS based on the alignment of 130 strains. Variation within each CDS was identified as a SNP or indel and counted with the use of an in-house Python script. Additionally, each SNP was characterized as synonymous or nonsynonymous. Nucleotide diversity values (*π*) for each CDS were calculated with TASSEL software (v.5.2.40; Bradbury et al., 2007). As nucleotide diversity is based only on nucleotide substitutions, the number of indels and percentage of polymorphic sites are given for each CDS. To reveal variation in intronic sequences, conserved introns (found in all studied strains) were extracted and analyzed as described for mitochondrial CDS.

## Results

### General Characteristics of Fungal Mitogenomes

Mitogenomes of species within the FGSC are highly conserved in terms of a set of 15 protein-coding genes, two rRNA genes (*rns* and *rnl*), and 28 tRNA genes, which were localized in the same order and orientation. Their mitogenomes displayed a similar GC content between 31.3 and 32.1%. However, mitogenome comparisons showed considerable differences in their size ranging from the smallest 88.409bp mitogenome of CBS 119183 *Fusarium cortaderiae* to the largest 106.714bp in CBS 119177 *F. vorosii*. This variation was mainly associated with a mosaic distribution of MGEs, which is described in one of the following sections.

**Figure 1:**
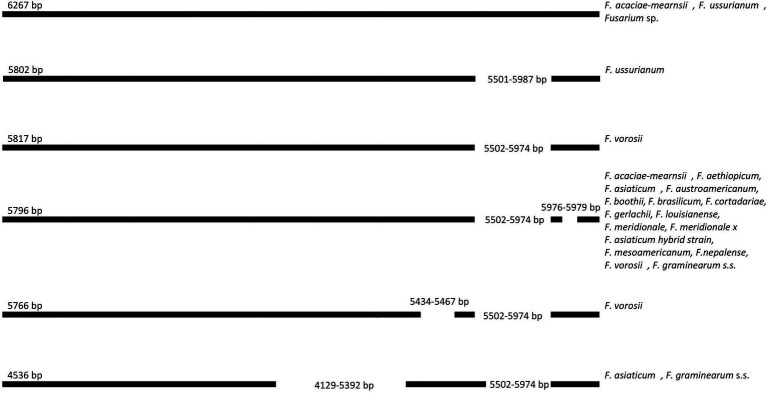
Schematic position of indels in LV-uORF in 122 *Fusarium* strains.

All FGSC strains contained a large open reading frame with unknown function (LV-uORF; Al-Reedy et al., 2012), which is in every strain located between the *rnl* and *nad2* genes. This LV-uORF differed in size from 4.536 to 6.273bp due to indel polymorphism (Figure 1).

### Mobile Genetic Elements and Their Distribution in FGSC

A total of 45 introns and 56 HEGs were identified in the set of mitochondrial protein-coding genes of the studied strains. Nineteen introns were highly conserved and present in all studied strains. More than half of the introns (*n*=24) identified were located in the three subunits of the cytochrome c oxidase (*cox* genes): 14 in *cox1*, 5 in *cox2*, and 5 in *cox3*. Positions of introns were highly conserved among different strains. All introns belonged to the group I intron family except i1 *cob* found in three strains: *F.g.* (INRA-156 and 16-462-z) and *F. vorosii* (CBS 119177), which was classified as belonging to the group II intron. This intron encoded a protein of the reverse transcriptase family. Most of group I introns harbored HEGs, of either the LAGLIDADG or GIY-YIG type, which can be determined based on differences in conserved amino acid motifs. Among 56 distinct HEGs, 33 were assigned as LAGLIDADG endonucleases and 16 as GIY-YIG endonucleases, while seven HEGs could not be precisely predicted as LAGLIDADG or GIY-YIG.

Eight introns showed very high similarity with no size difference variation, while 11 introns displayed some size variation due to small indels mostly below 100bp in size. Nine introns showed large size variation (the i4 *nad2* intron, the i1 and i2 *cox2* introns, the i2 and i5 *cob* introns, the i3 *cox1* intron, the i1 *atp6* intron, and the i1 and i3 *cox3* introns), mostly due to the irregular presence of HEGs. HEGs and/or introns that are absent in at least one studied strain are termed “optional” throughout this manuscript.

**Figure 2:**
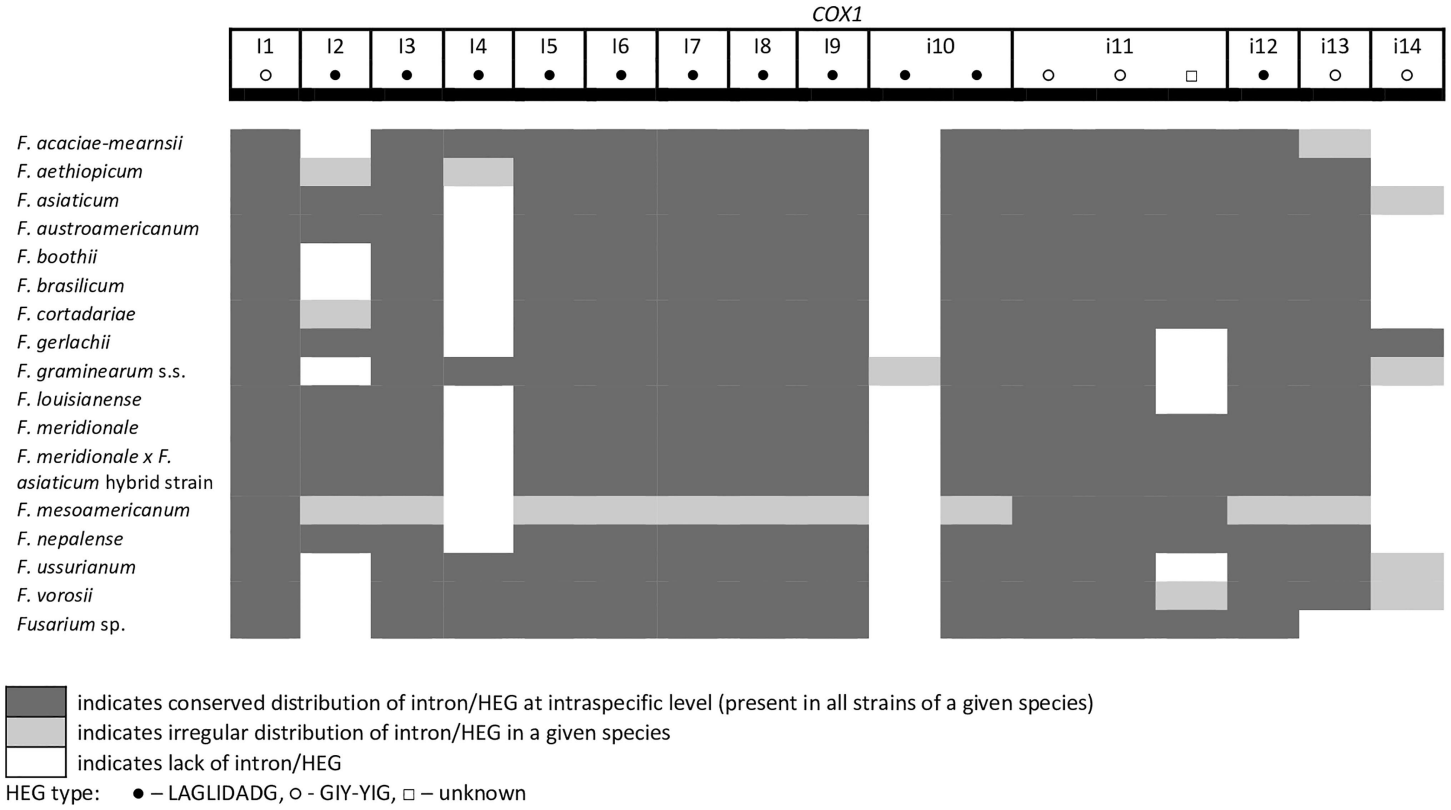
Distribution of introns and associated HEGs found in the *COX1* gene.

**Figure 3:**
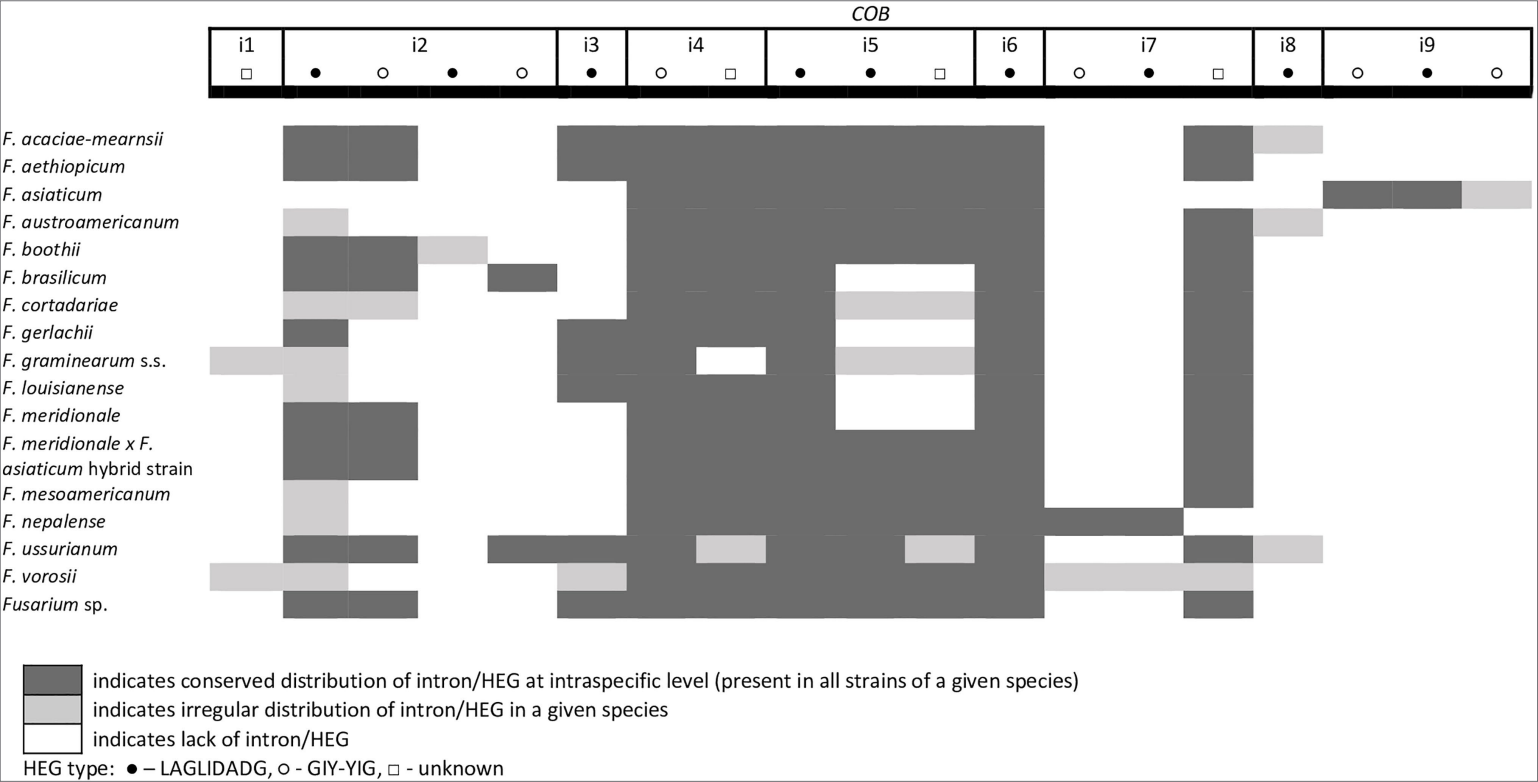
Distribution of introns and associated HEGs found in the *COB* gene.

**Figure 4:**
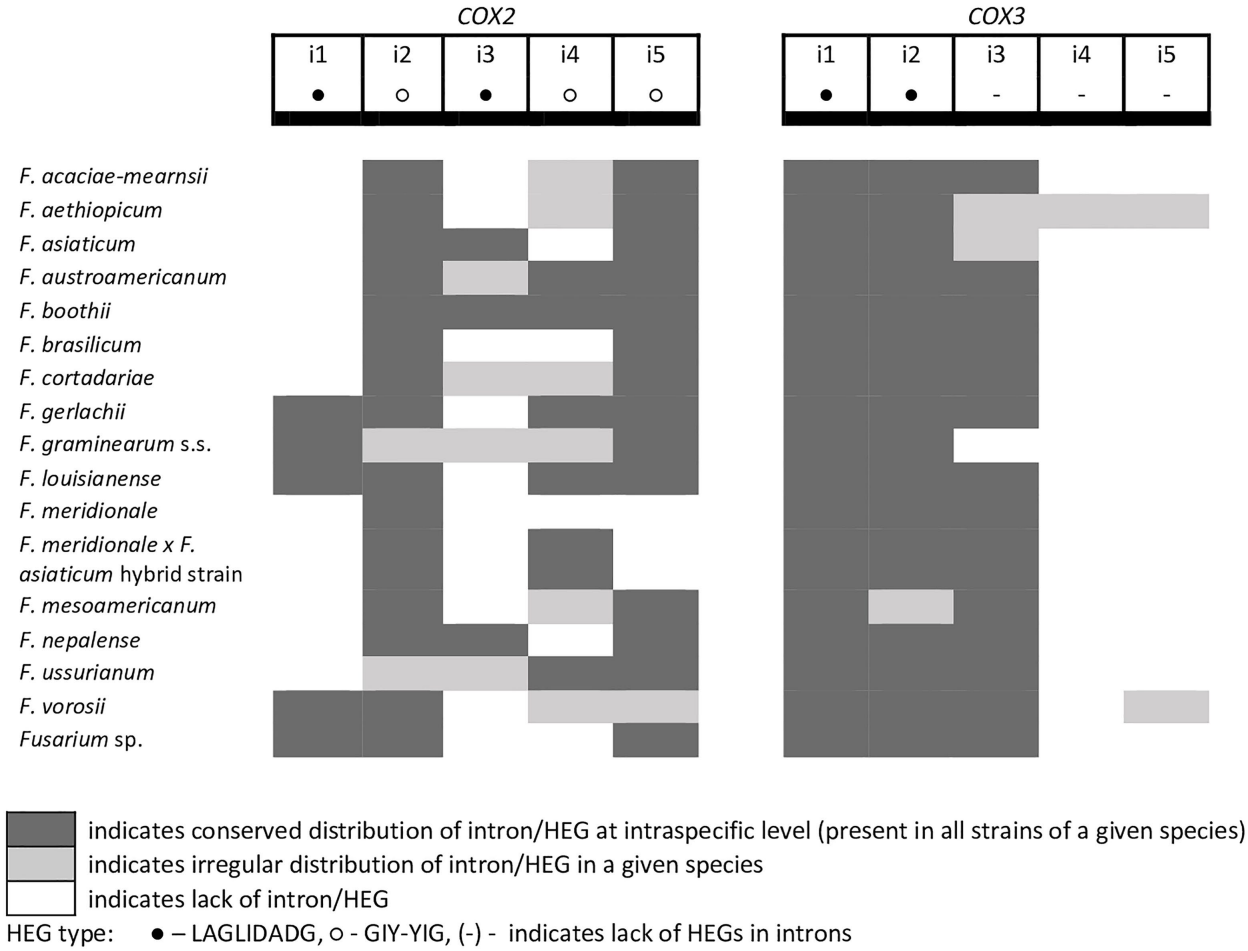
Distribution of introns and associated HEGs found in the *COX2* and *COX3* genes.

**Figure 5:**
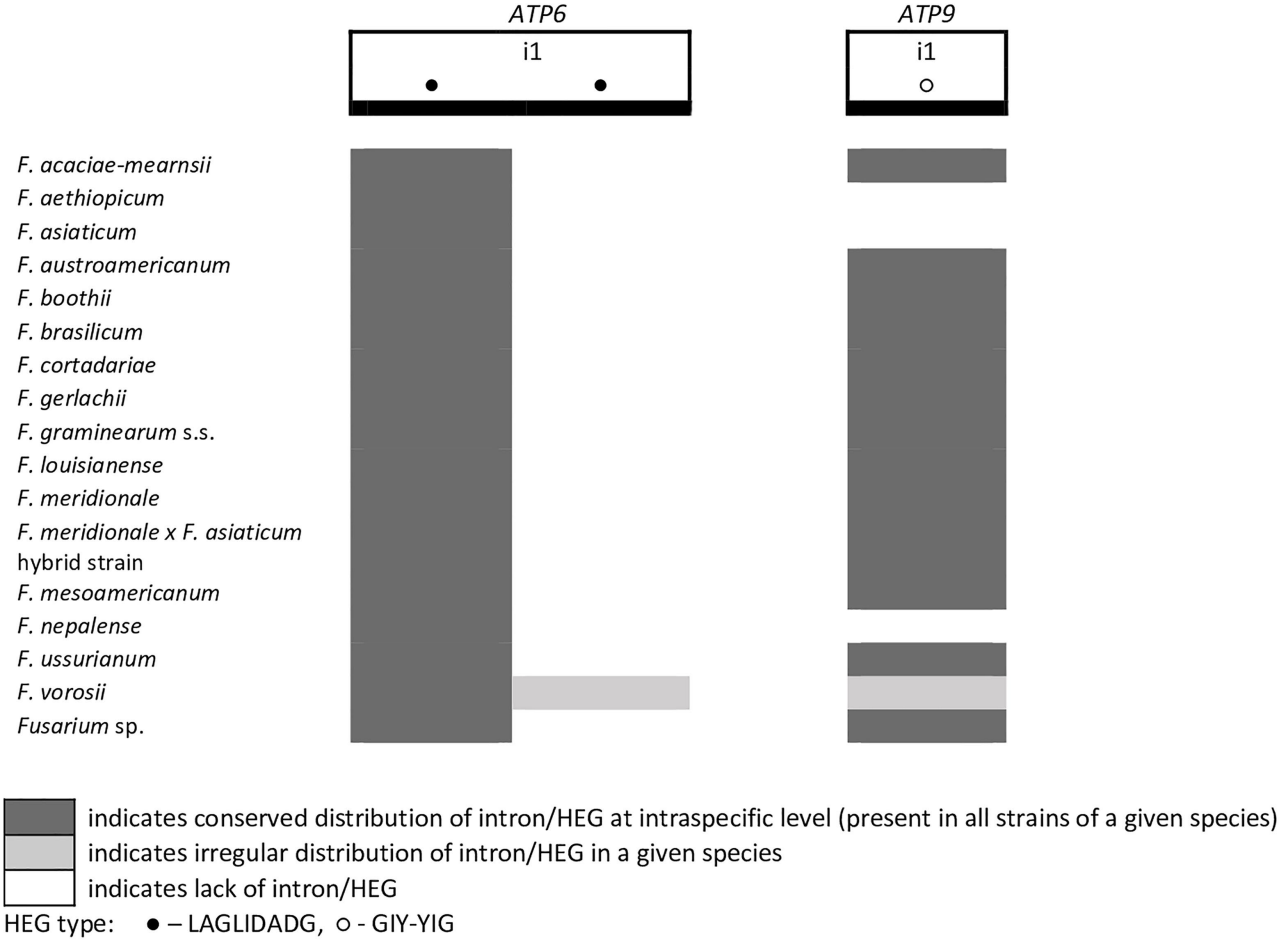
Distribution of introns and associated HEGs found in the *ATP6* and *ATP9* genes.

**Figure 6:**
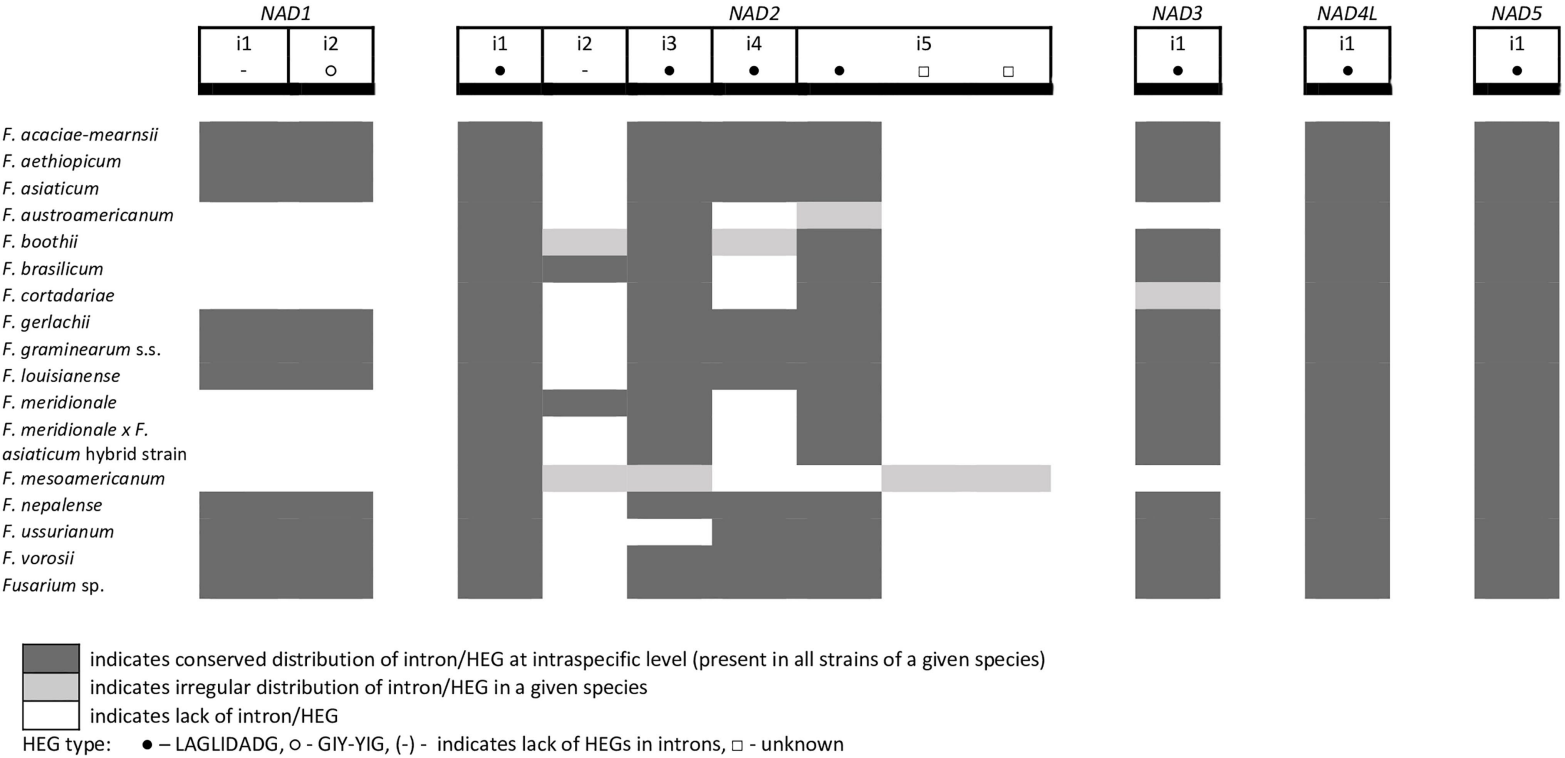
Distribution of introns and associated HEGs found in the *NAD* genes.

From the total 46 optional HEGs, 11 were very limited in distribution. A single strain of *F. mesoamericanum* (CBS 110252) harbored two optional HEGs (144 aa and 170 aa) in the i5 *nad2* intron. Due to loss of conserved amino acid motifs reflecting their functional differences, these two HEGs were not precisely determined to either LAGLIDADG or GIY-YIG endonucleases. Similarly, a single strain of *F. vorosii* (CBS 119177) contained an optional LAGLIDADG (352 aa) found in the i1 *atp6* intron. The presence of two optional GIY-YIG (314 aa and 315 aa) and one LAGLIDADG (196 aa) sequences in intron i9 of the *cob* gene was found in a single strain of *F. asiaticum* (CBS 110258). More frequent distributions were observed for the remaining MGEs found in the *cob* gene. An optional LAGLIDADG (474 aa) located in the i2 *cob* intron was shared by two (CBS 316.73 and CBS 119170) of the three strains of *F. boothii*. Three strains, CBS 123662 (*F. acaciae-mearnsii*), CBS 110244 (*F. austroamericanum*), and CBS 123754 (*F. ussurianum*), harbored an optional LAGLIDADG (234 aa) in the i8 *cob* intron. Two other HEGs found in the *cob* gene were distributed in FGSC at 12.5% frequency and included an optional GIY-YIG (127 aa) in the i2 *cob* intron of *F. brasilicum* and *F. ussurianum* and GIY-YIG (314 aa) as well as LAGLIDADG (192 aa) in the i7 *cob* intron of all strains of *F. nepalense* and *F. vorosii*. The remaining 24 optional HEGs (Supplementary File S1) were more commonly distributed, ranging in frequency from 21.9 to 97.1% (Supplementary File S1; Figures 2–6).

A large collection of *F.g.* strains enabled the study of MGE distribution in their mitogenomes and possible cryptic divergence. Forty different MGEs were found in *F.g.*; however, none showed a species-specific conservation. We previously found that a unique MGE pattern in the *cox3* gene allows reliable differentiation of *F.g.* from other closely related morphospecies. This unique pattern mainly concerns the species-specific loss of the i3 intron, which was evident in all strains of *F.g.*, regardless of their geographic origin. In this study, we found that in contrast to *F.g.*, the i3 *cox3* intron was present in almost all other members of the FGSC, except for two strains of *F. asiaticum* (191,317 and 180,363; Yang et al., 2020; Supplementary File S1). However, besides their irregular distribution, HEGs can differ in size due to small indel polymorphism (Table 1). To explore the value of this type of polymorphism for identification of *F.g.*, we compared a total of 1,564 individual HEGs and found that the difference in HEG size observed in eight HEGs (Table 1) can be used as a unique diagnostic feature for *F.g.*

**Table 1:** List of HEGs allowing differentiation of *F.g.s.s.* based on size difference.

Gene	Intron	HEG type	HEG size (nt)	
			*F.g.* *s.s.*	Other members of FGSC
*nad5*	i1 (IB)	LAGLIDADG	876	909
*cox2*	i5 (IC1)	GIY-YIG	1011	1242–1368
*cob*	i4 (ID)	GIY-YIG	642	870
*cob*	i7 (IC1)	unknown	1269	1419
*cox1*	i6 (IB)	LAGLIDADG	867	918
*cox1*	i7 (IB)	LAGLIDADG	957	972–1002
*cox1*	i8 (IB)	LAGLIDADG	1191	1221
*atp6*	i1 (IB)	LAGLIDADG	1035	1059

### SNP Analysis

We performed a whole-mitogenome SNP analysis to reveal the utility of this type of approach for differentiation of *F.g.* from the other species. We calculated the number of SNPs for every mitogenome to reveal if interspecific variability in mtDNA is greater than intraspecific variability, indicating the existence of the so-called “barcoding gap” in the data set. SNPs were detected from the core genome of assembled mitogenomes by multiple comparisons of studied mitogenomes against the reference mitogenome of PH-1 strain (CBS 123657, accession number: MH412632). Analyses of variation in both CDS and conserved introns (Tables 2 and 3) showed that most SNPs could be found in intronic sequences. In general, SNP variability was low, ranging from 9 to 230 SNPs per strain (Supplementary File S2). In most cases, intraspecific variability did not exceed 30 SNPs. However, two exceptional strains of *F.g.*, INRA-156 and CBS 134070, yielded 32 and 36 SNPs, respectively, which exceeded the least interspecies variability (31 SNPs) found for *F. vorosii* strain CBS 119177. Thus, assuming intraspecific variability below 30 SNPs, two out of 88 strains of *F.g.* could not be determined based on SNP analysis, which indicates 96% accuracy of this SNP-based approach. Besides exceptional strain (CBS 119177), the least interspecific variability was found for two strains of *F. gerlachii*, yielding 36 SNPs each. Surprisingly, the highest number of interspecific SNPs was found for two strains of *F. ussurianum* (227 and 230 SNPs, respectively), which exceeded the number of SNPs found in case of three morphospecies: *F. cerealis*, *F. culmorum*, and *F. pseudograminearum*. This result may indicate that some cryptic species may have an accelerated evolutionary rate with a unique evolutionary pattern in their mtDNA. In addition, the difference in evolutionary rate could be even observed at the strain level. Among the three strains of *F. vorosii*, CBS 119178 and CBS 123664 exhibited a similar number of interspecies SNPs (147 and 148 SNPs, respectively), which is in contrast to the exceptional strain (CBS 119177), which, as indicated above, yielded only 31 SNPs. An evidence for different evolutionary rates at the strain level was also observed by positioning some strains of the same species into scattered clades on a phylogenetic tree. This has been indicated in the next section.

**Table 2:** Variation in mitochondrial CDS among fungal strains.

Gene	Length (bp)	Synonymous SNPs	Nonsynonymous SNPs	Indels^*^	% PS	*π*
*atp8*	147	0	0	0	0	0
*atp9*	225	3	0	0	1.33	0.002
*nad4L*	270	1	0	0	0.37	0.00009
*nad3*	414	2	0	0	0.48	0.0005
*nad6*	681	0	0	0	0	0
*cox2*	750	5	0	0	0.67	0.0004
*atp6*	642–801	2	3	159	20.47	0.00324
*cox3*	810	25	8	0	4.20	0.00822
*nad1*	1110	0	0	0	0	0
*cob*	1173	2	0	0	0.17	0.00038
*nad4*	1455	2	0	0	0.14	0.00033
*rps3*	1482	1	2	0	0.20	0.00033
*cox1*	1593	31	0	0	1.95	0.00303
*nad2*	1665	2	1	0	0.18	0.00005
*nad5*	1989	1	1	0	0.10	0.00023

% PS, percent of polymorphic sites and π, nucleotide diversity values.

* Indels include single nucleotide insertions and deletions and deletion or duplication of longer tracts of DNA.

**Table 3:** Variation in conserved mitochondrial introns among fungal strains.

Intron name	Length (bp)	SNPs	Indels^*^	% PS	*π*
*cob i4*	2249	31	7	1.69	0.00193
*cob i5*	2467	16	1201	49.33	0.22581
*cob i6*	1061	7	0	0.66	0.00064
*cox1 i1*	1717	339	483	47.87	0.0293
*cox1 i3*	2398	449	1400	77.11	0.08436
*cox1 i4*	1375	9	0	0.65	0.00079
*cox1 i5*	1003	1	2	0.3	0.00027
*cox1 i6*	1154	6	45	4.42	0.00158
*cox1 i7*	1257	8	0	0.64	0.00026
*cox1 i8*	1325	476	275	56.68	0.00929
*cox1 i9*	2084	4	1078	51.92	0.00823
*cox1 i10*	2373	19	35	2.28	0.00595
*cox1 i11*	1198	1	0	0.08	0.00033
*cox3 i1*	1532	196	371	37.01	0.01505
*cox3 i2*	1875	682	499	62.99	0.01986
*atp6 i1*	3538	7	2086	59.16	0.00976
*nad2 i1*	1406	10	0	0.71	0.00073
*nad4L i1*	2008	34	208	12.05	0.01215
*nad5 i1*	1012	2	0	0.2	0.00014

% PS, percent of polymorphic sites and π, nucleotide diversity values.

* Indels include single nucleotide insertions and deletions and deletion or duplication of longer tracts of DNA.

### Phylogenetic Analyses

Phylogenetic trees of *atp9*, *cox1*, and *cox2* genes possess different topologies, which can be explained by their different evolutionary histories. In addition, majority of the well-supported clades were multispecies. These results arise from low CDS variation in the studied strains, which were further confirmed by calculating nucleotide diversity values, number of polymorphic sites, and SNPs (Table 2). We also found that some strains of the same species were scattered in different clades of the tree. This was mostly evident in the *cox1* tree for strains from *F.g.* and other cryptic species such as *F. boothii*, *F. mesoamericanum*, *F. cortadariae*, and *F. vorosii* (Supplementary File S4), suggesting incomplete lineage sorting and introgression in the course of the evolution of the studied Fusaria.

## Discussion

The introduction of molecular tools has revolutionized the diagnostics of microorganisms, including fungal plant pathogens. The use of DNA-based markers opens possibilities for improved control of specific pathogens and pathotypes (Luchi et al., 2020). Identification of fungi based on mtDNA provides a valuable alternative to existing molecular approaches based on nuclear data, especially when efficient recovery of DNA is problematic. mtDNA can be amplified with higher success than nuclear DNA due to its high copy number in fungal cells (Krimitzas et al., 2013; Franco et al., 2017). However, exploration of mtDNA for diagnostic purposes may be challenging due to difficulties in detecting species-specific polymorphism. These difficulties are mostly reflected by (1) the highly conserved nature of mtDNA (Zhang et al., 2020), (2) intrastrain mosaicism in MGE patterns (Losada et al., 2014), and (3) phylogenetic discordance between different mitochondrial genes (Brankovics et al., 2017; Fourie et al., 2018). A previous comparative analysis of a large set of *F.g.* strains underlined the high conservation and similarity of mitogenomes in closely related morphospecies: *F. cerealis*, *F. culmorum*, and *F. pseudograminearum*. In general, mitogenomes of these closely related species were MGE rich, and their irregular distribution among strains determines, to a large extent, mitogenome variation. MGEs that do not display mobility among other lineages can be fixed in species. Such targets are good candidates for developing diagnostic tools for identification of species; however, their identification requires comparison of a large set of target and nontarget strains. On the other hand, frequent mobility of MGEs could be incorporated into population genetic studies illustrating the local genetic variation and gene flow (Wolters et al., 2015; Wu et al., 2019).

In this study, we confirmed that the irregular distribution of MGEs does not follow divergence of cryptic species within the FGSC. This finding supports the hypothesis of low mobility of ancestrally acquired MGEs that are consequently shared by geographically diverse cryptic species (Kulik et al., 2020b). These ancestral elements may undergo changes throughout evolution, and assuming the lack of horizontal gene flow, these alternations should, at least in part, exhibit species-specific polymorphism. Indeed, in this study, we found that HEGs shared by different species can display small indel polymorphism that is species specific. The small differences in lengths of certain HEGs (Table 1) may facilitate further differentiation of *F.g.* from the other members of the FGSC.

In this study, we also explored the value of SNP analysis for typing *F.g.* This approach is increasingly used in the epidemiologic analyses of bacterial (Schürch et al., 2018) and fungal pathogens (Uelze et al., 2020b); however, to our knowledge, it has not yet been tested using fungal mtDNA. Successful identification of strains to the species level is achieved based on difference in intraspecific and interspecific SNP divergences (Bae et al., 2021). We found that intraspecific variation among *F.g.* strains was very low. Based on our assumption that intraspecific variation may not exceed 30 SNPs, the success rate in identifying strains of *F.g.* was estimated at 96%. The high confidence of this approach, its simplicity to perform, and the limited time needed for the analysis make this tool an attractive complement to other more refined techniques for identifying of *F.g.* (Wu et al., 2019; Kulik et al., 2020b).

Mitochondrial sequences can contribute to species identification due to their efficacy in revealing phylogenetic relationships among the studied taxa. However, we found that for *F.g.* and the other cryptic species within the FGSC, this approach is not discriminative enough mostly due to the highly conserved nature of mtDNA and lack of concordance between the different gene genealogies, which presumably results from incomplete ancestral lineage sorting. Our findings on the lack of concordance between different phylogenies of mitochondrial genes are in line with previous studies by Fourie et al. (2013, 2018) and Brankovics et al. (2020) who detected interspecies gene flow among mitogenomes of closely related members of the FFSC and FOSC. The success in detecting ancestral gene flow events through mitochondrial comparisons is due to the greater levels of conservation and synteny of fungal mitogenomes than observed in nuclear compartments (Brankovics et al., 2020). However, from a diagnostic point of view, this phylogenetic discordance largely limits clustering-based identification approaches (Wu et al., 2018).

Overall, the results presented in this study showed that the use of mtDNA provides valuable information for identification of cryptic species within fungal complexes. Although fungal mitogenomes lack a “universal barcode” for tagging cryptic species, they might display other patterns of species-specific conservation. Uncovering of these sites requires testing a large collection of geographically diverse strains to differentiate between strain- and species-specific variation.

## Conclusion

Mitogenomes show promise for identification purposes of important cryptic species like *F.g.* in the FGSC. Improved identification could be achieved by the combination of intronic variation analysis and whole-mitogenome SNP analysis. However, mitogenomes also show evidence of ancestral gene flow among other members of the FGSC, which largely limits clustering-based identification.

## Data Availability Statement

The datasets presented in this study can be found in online repositories. The names of the repository/repositories and accession number(s) can be found in the article/Supplementary Material.

## Author Contributions

JW and TK wrote the manuscript. JW, AD, MŻ, AS, and KB contributed to isolation and strain growth. JW, MŻ, and KB extracted the fungal DNA and prepared libraries for whole-genome sequencing. TK assembled the mitogenomes and was responsible for the study design. KM and TM performed the mitogenome annotation. TM performed the phylogenetic analysis. AD and AS edited the manuscript. All authors contributed to the article and approved the submitted version.

## Conflict of Interest

The authors declare that the research was conducted in the absence of any commercial or financial relationships that could be construed as a potential conflict of interest.

## Publisher’s Note

All claims expressed in this article are solely those of the authors and do not necessarily represent those of their affiliated organizations, or those of the publisher, the editors and the reviewers. Any product that may be evaluated in this article, or claim that may be made by its manufacturer, is not guaranteed or endorsed by the publisher.

## Footnotes

^1^ https://www.edgebioinformatics.org/
