# Supplementary figures and images for: Uncovering Diagnostic Value of Mitogenome for Identification of Cryptic Species Fusarium graminearum Sensu Stricto
Source: Front Microbiol. 2021 Aug 31;12:714651. doi: 10.3389/fmicb.2021.714651 (PMC8439580; doi:10.3389/fmicb.2021.714651)

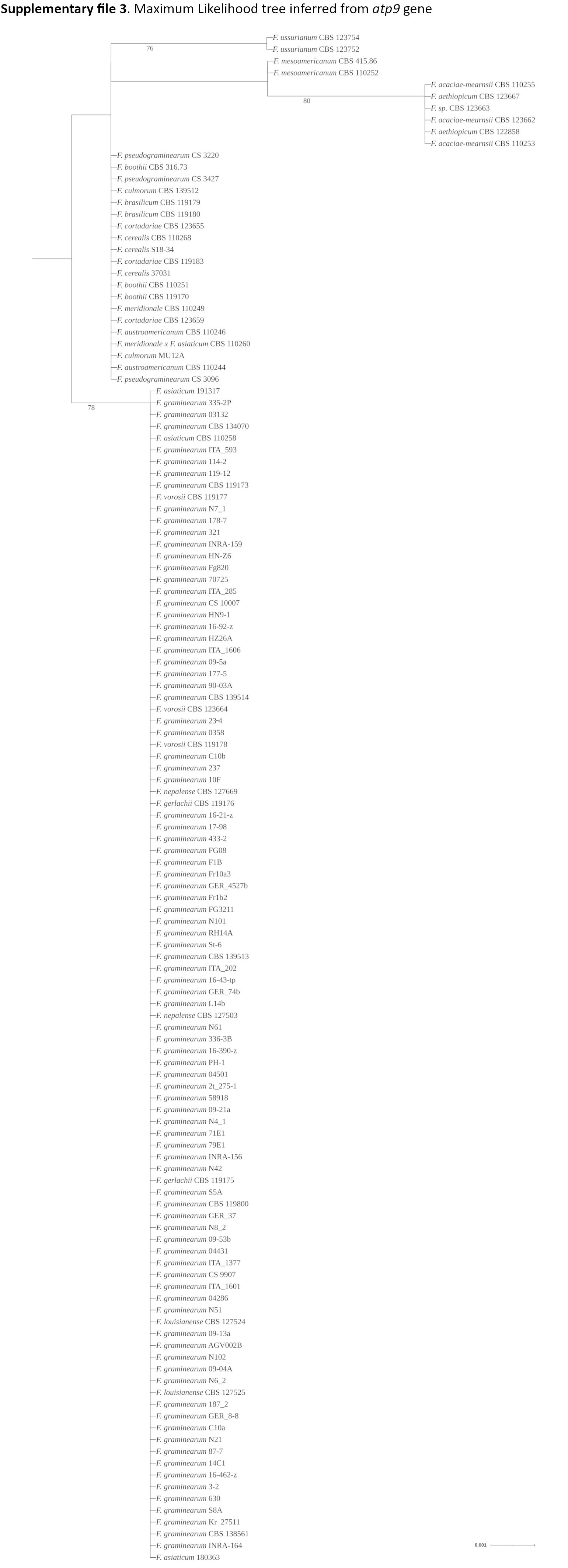

Supplement: Supplementary file 1 [file Image_1.PNG]

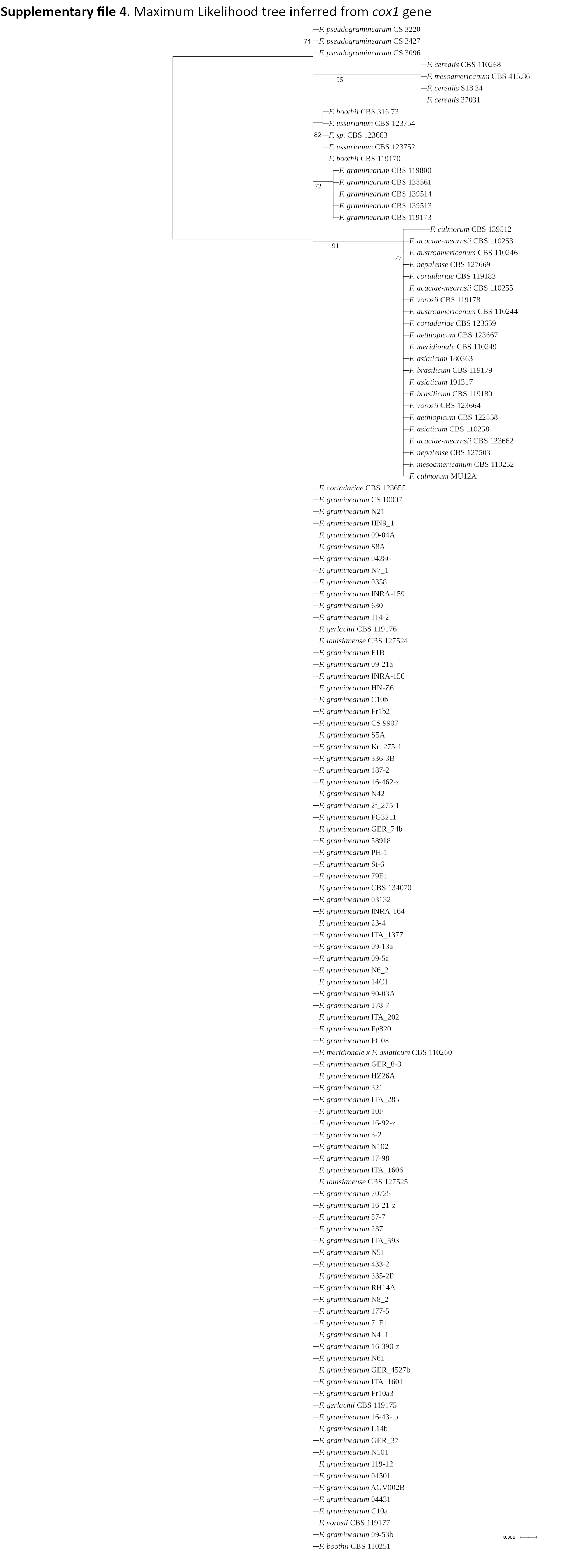

Supplement: Supplementary file 2 [file Image_2.PNG]

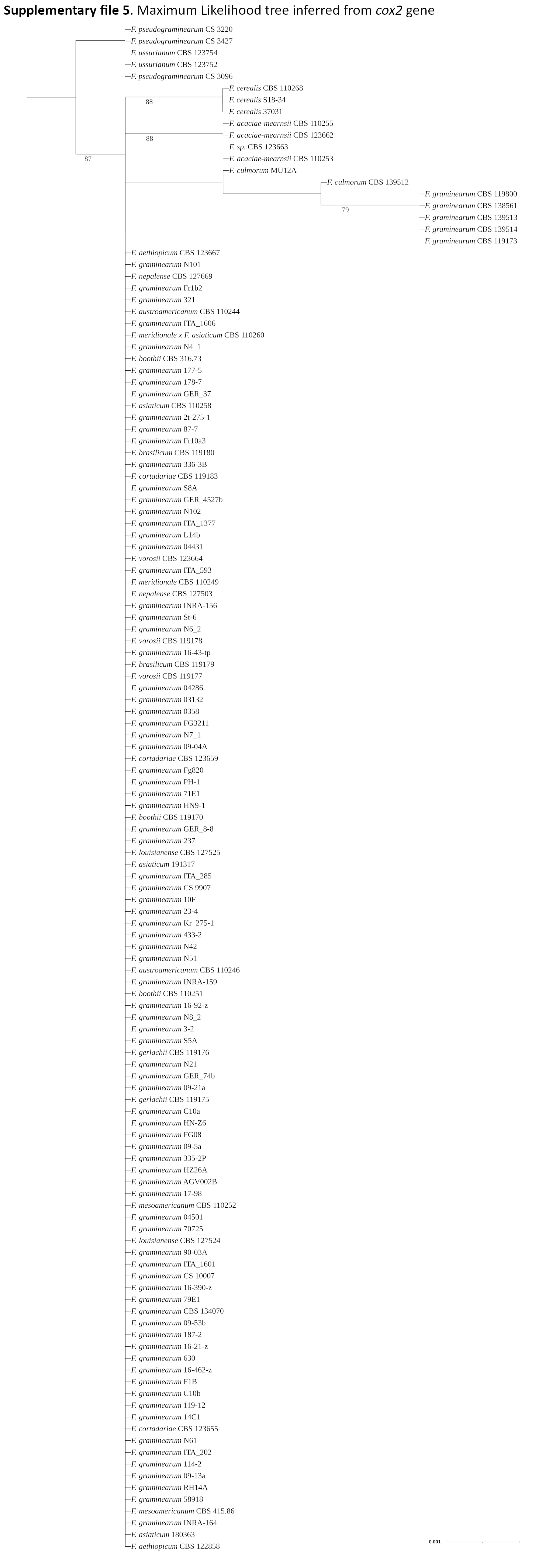

Supplement: Supplementary file 3 [file Image_3.PNG]
